# UV-LIGA Microfabrication for 1.1 THz Staggered Double-Grating Slow-Wave Structures

**DOI:** 10.3390/mi17040427

**Published:** 2026-03-31

**Authors:** Qi Jiang, Xinghui Li, Yuanfei Hui, Pan Pan, Jinjun Feng

**Affiliations:** National Key Laboratory of Science and Technology on Vacuum Electronics, Beijing Vacuum Electronics Research Institute, Beijing 100015, China

**Keywords:** micro-electro-mechanical system (MEMS), staggered double-grating, slow-wave structure (SWS), UV-LIGA

## Abstract

In this paper, a 1.1 THz staggered double-grating slow-wave structure (SWS) for traveling wave tubes (TWTs) is fabricated using UV-LIGA processes based on RD-2150 photoresist. The designed SWS has a wide side of 170 μm and a narrow side of 50 μm, and when half of the SWS is fabricated, the height of the structure is 85 μm, which is half of the wide side. The fabrication process includes lithography, electroforming, grinding, polishing, and resist removal. The top surface, bottom surface, and sidewall roughness of the as-fabricated structure were measured to be 21 nm, 20 nm, and 17 nm. The mean measured sidewall verticality of the structure was 90.1°, with a standard deviation of 0.5° obtained from four independent sampling positions. For the structure’s nominal dimensions of 85 μm in height and 50 μm in width, the achieved dimensional accuracies were ±2 μm and ±1 μm, with corresponding standard deviations of 1.05 μm and 0.59 μm, respectively, confirming excellent structural uniformity. We subsequently evaluated the impact of these dimensional deviations on the electromagnetic performance of the structure. The results indicate that the deviations had a negligible effect on the dispersion characteristics. Specifically, the linewidth deviation led to a 21% reduction in coupling impedance, while the height deviation caused a 600 V increase in the synchronous operating voltage.

## 1. Introduction

High-Frequency vacuum electron devices have great potential in radar [1], communications [2,3], and scientific research [4]. Traveling wave tubes (TWTs), characterized by high output power and a wide operating bandwidth, are widely used in high-power millimeter-wave amplification devices [5]. TWTs consist of electron guns, magnetic focusing field systems, slow-wave structures (SWSs), attenuators, energy transmission windows, and collectors. In this paper, we fabricated an SWS for 1.1 THz TWTs using UV-LIGA processes. The size of the SWS in this frequency is only tens of microns and even with the most advanced CNC milling, it is difficult to ensure high quality and repeatability. Only lithography can fabricate structures with higher dimensional accuracy and lower surface roughness [6,7,8]. Furthermore, microfabrication technology can meet the requirements of small-scale mass production. Compared to Deep Reactive Ion Etching (DRIE), the UV-LIGA process offers precise lithography and copper electroforming capabilities [9,10,11], meeting the requirements for vacuum-compatible, all-copper circuit microfabrication. The all-copper structure fabricated by UV-LIGA can ensure low microwave loss and high thermal conductivity at high frequencies [12,13], solving the problems of silicon composite structures, such as film detachment and poor heat conduction. H. Li and J. Feng et al. have systematically developed UV-LIGA fabrication technology for high-precision folded waveguides (FWGs) covering W-band to 340 GHz. In their initial work, they demonstrated W-band FWGs with dimensional accuracies of ±2 µm in height and ±10 µm in width [14,15]. They subsequently optimized the process to fabricate 220 GHz FWGs, achieving a height accuracy of 10 µm and a width accuracy of 6 µm [16]. With further process refinement, the team realized 340 GHz FWGs with improved precision, where the height and width dimensional accuracies were controlled to below 6 µm and 2 µm, respectively [17]. In addition to slow-wave structures, the all-metal UV-LIGA process is also suitable for the precision fabrication of structures such as transition waveguides. Y. Li et al. fabricated a WR2.8 terahertz rectangular waveguide, and the accuracy of the height and width was 5 µm and 2 µm, respectively [18].

To our best knowledge, SWSs above 1 THz have only been reported by DRIE fabrication [19]. For the first time, we have extended the UV-LIGA process to the fabrication of 1.1 THz SWSs for TWTs and optimized the dimensional accuracy and uniformity. Structural uniformity represents a key challenge in high-frequency terahertz device manufacturing, as it requires precise periodic replication of slow-wave structures (SWSs) to meet the full Floquet boundary conditions. Focusing on the fabrication of 1.1 THz all-metal SWSs, this work has realized a breakthrough in dimensional accuracy, with fabricated samples outperforming previously reported devices in precision. In addition, the comprehensive characterization of structural uniformity is performed, verifying that the achieved precision is not a single-point optimal value, but is maintained uniformly across the entire SWS samples. The height of the waveguide is jointly determined by the substrate and the polished plane. The width and verticality of the sidewalls are determined by lithography. The roughness is determined by lithography and electroforming. The main challenges include: constructing a perfectly flat substrate plane to avoid UV light scattering on the metal surface during lithography; constructing a flat photoresist plane to avoid diffraction during lithography; and constructing a flat grinding and polishing plane to ensure consistency within the structure.

## 2. Designing Variables

**Figure 1:**
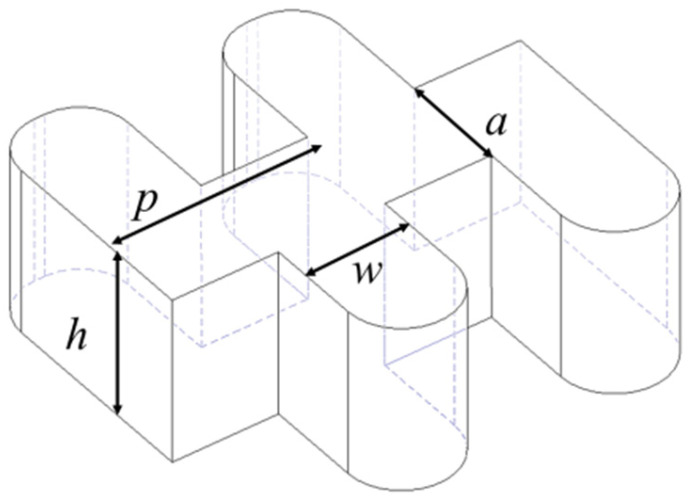
Schematic dimension diagram of the 1.1 THz staggered double-grating SWS.

**Figure 2:**
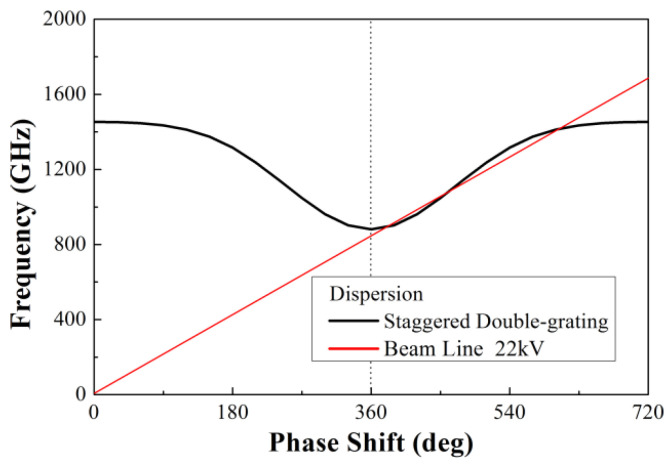
The Brillouin dispersion characteristics of the structure.

**Figure 3:**
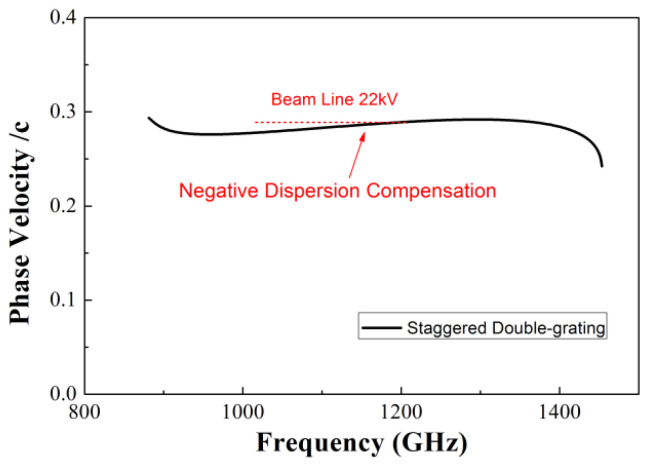
Phase velocity versus beam voltage curve.

**Figure 4:**
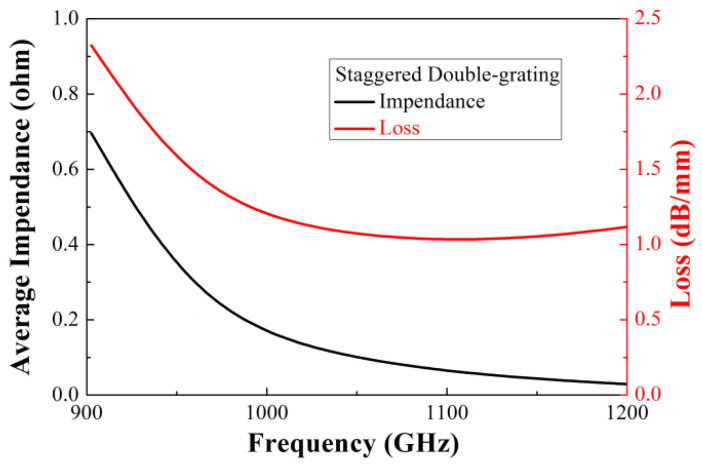
Coupling impedance and ohmic loss curves.

We present the staggered double-grating SWSs for 1.1 THz TWTs. The staggered double-grating SWS is a two-dimensional planar periodic configuration with a natural electron beam tunnel, compatible with micromachining and easy for batch production. Figure 1 shows two periods of the structure, where *h* and *w* are the height and width of the waveguide, respectively, *p* denotes the beam path lengths in one individual period, and *a* denotes the width of the electron beam tunnel. Figure 2 shows the Brillouin dispersion characteristics of the structure. The dispersion curve intersects the 22 kV beam voltage line near 1.1 THz, which can be clearly observed in Figure 3. Benefiting from the design of relatively flat anomalous dispersion, the beam voltage line achieves better synchronization matching at the high-frequency band. This design is intended to compensate for the gain drop caused by the low coupling impedance in the high-frequency band, and this dispersion characteristic enables more consistent in-band gain. Figure 4 presents the average coupling impedance and ohmic loss of the designed staggered double-grating structure. The structure has a coupling impedance of about 0.1 Ω and a loss of about 1 dB/mm, where the equivalent conductivity of oxygen-free copper (OFC) is set to 1.6 × 10^7^ S/m.

The dimensions of the 1.1 THz staggered double-grating SWS are listed in Table 1.

## 3. Fabrication

**Figure 5:**
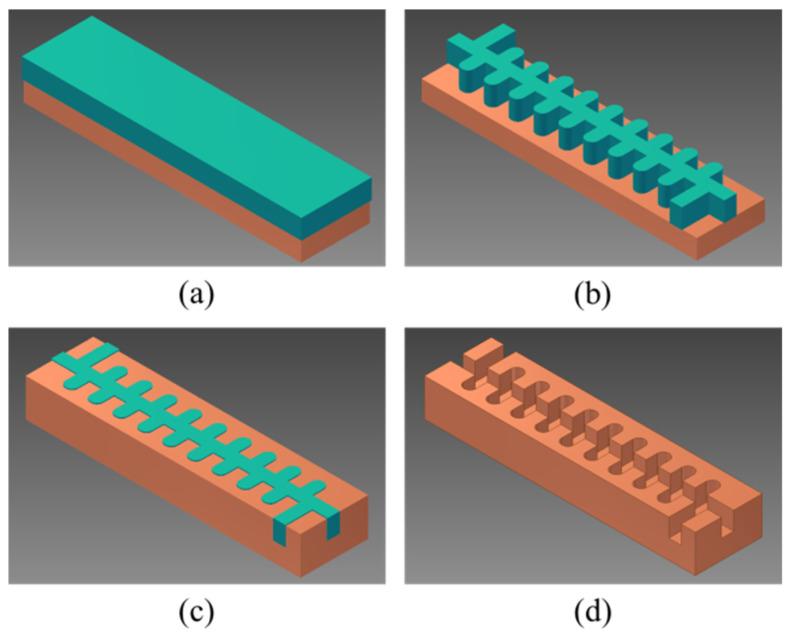
Flowchart of UV-LIGA fabricating technology. (**a**) Coating of photoresist. (**b**) Exposure and development. (**c**) Electroplating, grinding, and polishing. (**d**) Removing of the photoresist.

The flowchart of the 1.1 THz staggered double-grating SWSs by UV-LIGA is shown in Figure 5. First, a layer of RD-2150 photoresist was coated onto the substrate, followed by soft baking on a flat hotplate. RD-2150 is a negative photoresist similar to SU-8 with epoxy resin as its main component, and was purchased from Suzhou Yancai Micro-Nano Co., Ltd., Suzhou, China. Under the shielding of the mask with staggered double-grating patterns, the sample was exposed to 365 nm ultraviolet light, followed by a post-bake. After development, the photoresist layer with a reversed pattern was used as a mold to deposit copper by electroforming, where copper layers were grown in the non-photoresist areas of the substrate. After grinding and polishing to the target height, the photoresist was removed to obtain the final half-waveguide.

### 3.1. Substrate Pretreatment

The substrate used in UV-LIGA ultimately serves as a part of the SWS. It requires high flatness, high smoothness, and a low oxygen content to ensure an accurate lithography, a consistent height of the structure, a low device loss, and a stable annealing process.

In the experiment, we used a 4-inch oxygen-free copper substrate with a thickness of 10 mm. Firstly, the substrate was subjected to 850 °C hydrogen annealing to release internal stress. Due to the irregular grain growth and rearrangement that occur during the high-temperature process, the flatness of the substrate decreases. After annealing, both sides of the substrate were reground and repolished to ensure a surface roughness of <20 nm and a flatness of <5 μm. The polished substrate was ultrasonically cleaned in acetone for 20 min to remove oil, soaked in hydrochloric acid for 30 s to remove the oxide layer, rinsed with anhydrous ethanol to remove residual chloride ions on the substrate, and finally dried in an N_2_ stream.

### 3.2. Photoresist Coating, Soft Bake, and Exposure

**Figure 6:**
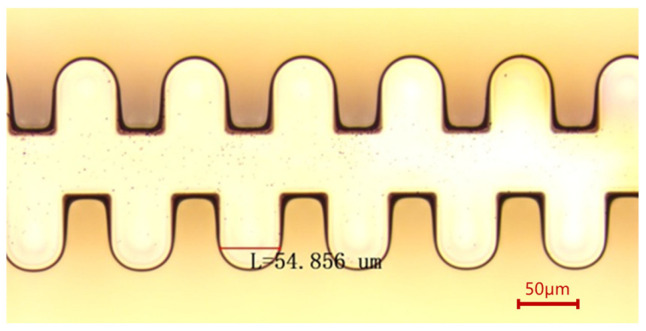
Dimensional deviation caused by the non-uniformity of the photoresist thickness. The design value is 50 μm.

The photoresist used in the UV-LIGA process has low fluidity and high viscosity, making it difficult to be uniformly adhered via spin coating. After surface coating, gravity self-leveling was generally used to flatten the photoresist. The photoresist thickness for the 1.1 THz SWSs is approximately 200 μm, resulting in a non-uniformity of about 30 μm because the gravity leveling effect worsened. This created a large gap between the mask and the resist in some areas during lithography, causing dimensional deviation, as shown in Figure 6.

**Figure 7:**
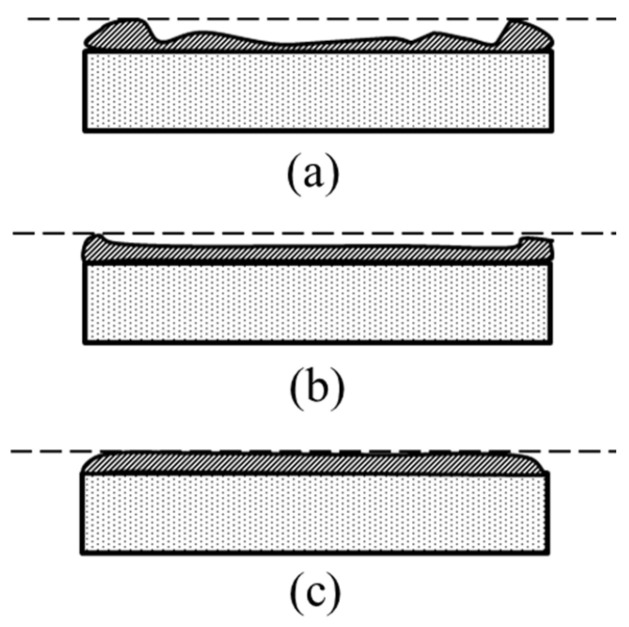
Flowchart of photoresist coating for 1.1 THz SWSs. (**a**) Coating of photoresist. (**b**) Fixed-position doctor blade processing. (**c**) Blading the edge resist. The dotted line represents the position of the mask in the subsequent lithography stage.

**Figure 8:**
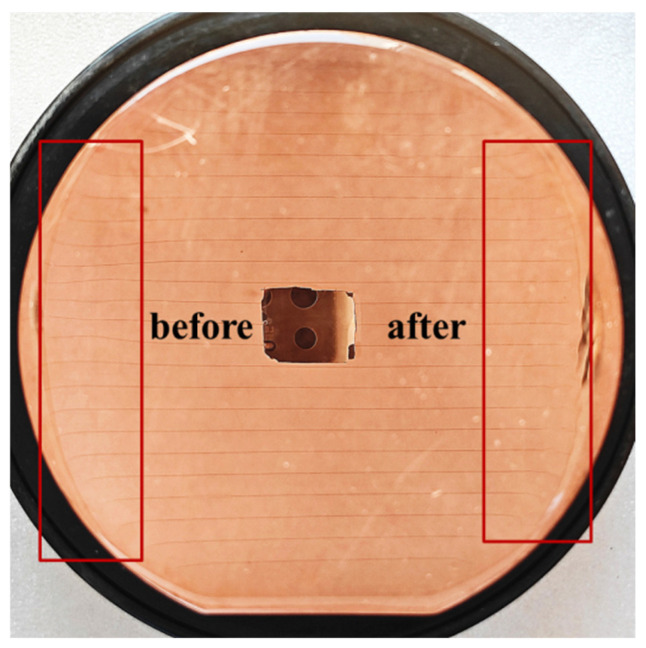
The effect of edge leveling. The lines are a reflection of the light from a wide-ruled notebook paper to clarify the defects.

**Figure 9:**
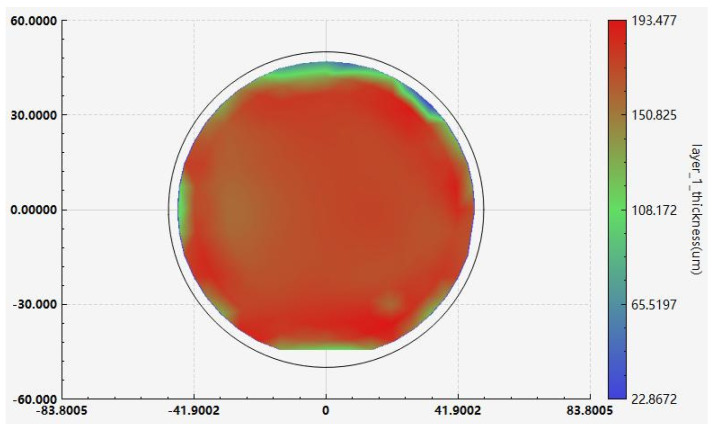
Photoresist thickness distribution.

We added two extra steps to the previous coating method. The flowchart of the new coating process is shown in Figure 7. We designed a fixed-position horizontal doctor blade platform. First, photoresist thicker than the target thickness was coated on the copper substrate, as shown in Figure 7a. Then, the substrate was placed under the horizontal doctor blade, which was set 200 μm above the substrate plane, and was passed through the blade with the traversing speed of the doctor blade set to 1 mm/s. This step levels the photoresist in the central areas of the substrate. Due to surface tension, the edge bead effect occurred, resulting in a film thickness at the edges exceeding the target value, as shown in Figure 7b. Next, another blade was applied at the substrate edges to scrape off the resist, controlling the resist height at a position about 2 mm from the edge to be lower than the target value. This ensures close contact between the mask and the photoresist in the effective pattern area during contact exposure, as shown in Figure 7c. After this operation, the pre-bake time needs to be extended to compensate for the film deformation caused by blading. Our soft bake conditions were held at 65 °C for 15 min, then we raised the temperature to 95 °C for 100 min. The hotplate was subsequently turned off, and the resist was left to cool to room temperature. The effect of edge leveling in the new coating method is shown in Figure 8. We conducted a comparative test on the same copper sample, and the only difference was whether the step illustrated in Figure 7c was implemented. The sample was observed using reflected light from a wide-ruled notebook sheet. It is obvious that when the edges were untreated, the non-uniformity of the edge resist caused the parallel stripes to be distorted. After edge treatment, the degree of stripe distortion was reduced, and the flatness was significantly improved. The uniformity of approximately 200 μm resist improved from over 30 μm to within 10 μm in the effective patterned area of a 4-inch substrate. Figure 9 shows the photoresist thickness distribution obtained with this method.

After soft bake, the RD-2150 resist was exposed to UV light with a wavelength of 365 nm on a contact lithography exposure machine. An appropriate exposure dose is one of the most critical parameters affecting the dimensions of patterns. Through the optimization of photoresist uniformity in the previous steps and appropriate dose selection, we obtained results with a linewidth deviation within ±1 μm. The optimized exposure dose was 375 mJ/cm^2^ at an intensity of 15 mW/cm^2^. During exposure, soft-contact exposure and intermittent exposure were adopted. Every single exposure lasted 2.5 s, followed by a 10-s interval before the next exposure cycle, with a total of 10 exposure cycles.

### 3.3. Post-Exposure Bake and Development

We baked the sample on the hotplate to crosslink the epoxy resin after exposure, transforming the photolithography pattern from a soft crosslinked state to a hard, chemically stable solid structure. Due to the difference in thermal expansion coefficients between the copper substrate and the photoresist, an excessively high post-exposure bake temperature tends to cause resist detachment, while insufficient temperature leads to inadequate crosslinking. We studied different post-exposure bake conditions and developed the samples. The resist was baked by ramping the temperature from 35 °C to 55 °C for more than 10 min, keeping the temperature of the hotplate at 55 °C for 10 min. Then, the temperature was raised to the maximum temperature for 25 min, followed by natural cooling to room temperature.

**Figure 10:**
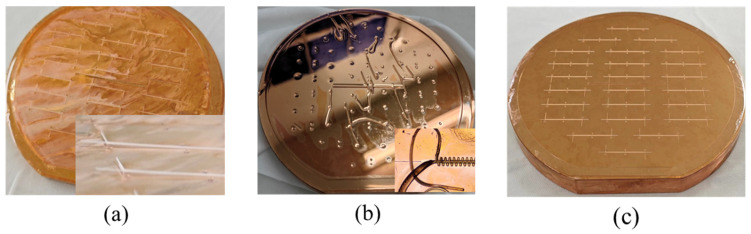
Development results obtained by various maximum temperatures in post-exposure bakes. (**a**) 95 °C; (**b**) 65 °C; and (**c**) 75 °C.

When the maximum temperature was set to 95 °C, the photoresist was fully crosslinked but exhibited excessive stress, leading to “hard detachment” during development, as shown in Figure 10a. The photoresist pattern was rigid but warped and fell off the substrate. When the maximum temperature was set to 65 °C, insufficient crosslinking caused swelling of the photoresist and the collapse of the pattern, as shown in Figure 10b. When the maximum temperature was set to 75 °C, the photoresist adhered perfectly to the copper substrate, as shown in Figure 10c.

In the above process, development was performed using MicroChem’s SU-8 developer purchased from Suzhou Yancai Micro-Nano Co., Ltd., Suzhou, China. First, the sample was inverted in the developer and left static for 5 min. Then, megasonic development was used for 30–60 s to thoroughly clear the photoresist from the structural gaps. The sample was rinsed sequentially with isopropyl alcohol and deionized water, and then dried with an N_2_ stream.

### 3.4. Electroforming, Grinding, and Polishing

**Figure 11:**
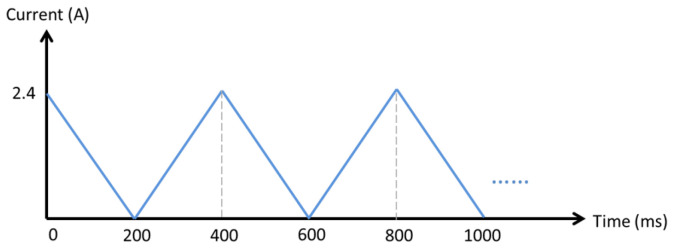
Waveform of triangular-wave electroforming.

The electroforming cathode was the 4-inch copper sample with the staggered double-grating photoresist pattern. The anode consisted of many small copper balls held in a pocket. The electroforming bath contained copper sulfate, sulfuric acid, chloride ions, and additives. Triangular wave electroforming was used at a temperature of 25 °C, with an average current of 0.6 A, and a duty cycle of 50%. The electroforming waveform is shown in Figure 11. The growth rate was controlled at approximately 15 μm/h for 12 h. During the process, the electroforming bath was continuously circulated.

**Figure 12:**
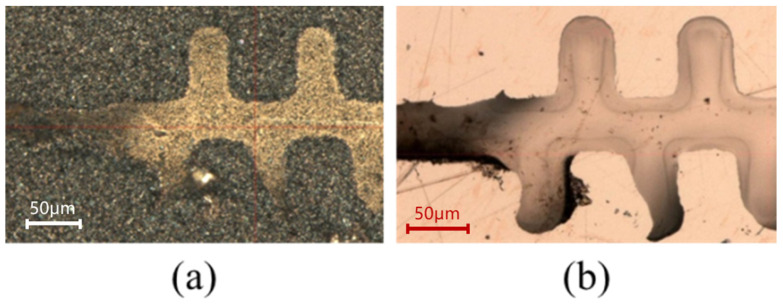
The structural damage caused by abrasive scratches. (**a**) The scratch damage generated during the grinding process. (**b**) The scratch damage cannot be repaired by the polishing process.

The sample was then ground and polished down to the target height using a PM5 grinder/polisher from Logitech Limited (Scotland, UK). We designed two mirror-image structures on the same substrate. The SWS is formed by bonding two mirrored half-waveguides, which require good height uniformity and consistency. The width of the 1.1 THz structure was only 50μm, and the scratches generated during the grinding process have a significant impact on the structural morphology. Scratches that cannot be eliminated in the subsequent polishing process will directly lead to structural damage, as shown in Figure 12. We sequentially used alumina abrasives with particle sizes of 20 μm, 9 μm, and 3 μm, which helped reduce the structural damage caused by abrasive scratches. First, 20 μm coarse abrasive particles were used for planarization at a rotational speed of 80 rpm and a droplet feed rate of one drop per second, and the grinding process was continued until the entire surface of the sample was visually confirmed to be uniformly abraded. The abrasive was then replaced with 9 μm particles to repair the damage layer caused by the previous coarse abrasive, with the rotational speed kept at 80 rpm and the feed rate at one drop per second, and the process was stopped after approximately 30 μm of copper was removed. Finally, 3 μm abrasive was employed to further eliminate the residual damage layer from the 9 μm grinding step; owing to the ultra-fine particle size and the narrow gap between the sample and the polishing pad, surface scratches were likely to occur, so the rotational speed was reduced to 60 rpm and the feed rate was adjusted to three drops per second to suppress scratch formation, and the grinding process was finished when the central region of the sample was 15 μm above the target height and the edge region was 10 μm above the target height.

**Figure 13:**
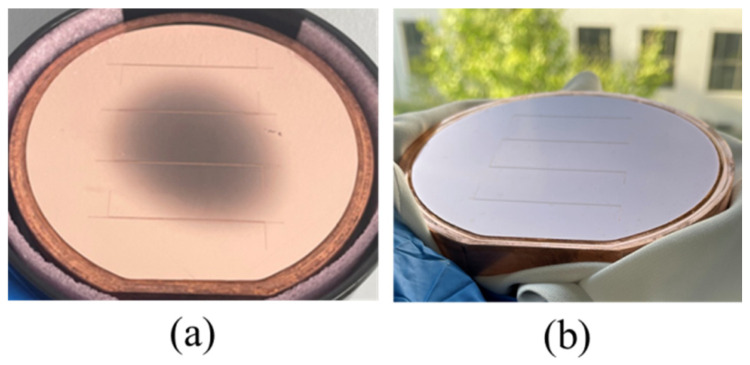
The effect of a 5 μm reserved height in the central region on the polishing process. (**a**) No reserved height, distinct brightness boundary forms between the central and edge regions. (**b**) With reserved height, the sample brightens uniformly.

**Figure 14:**
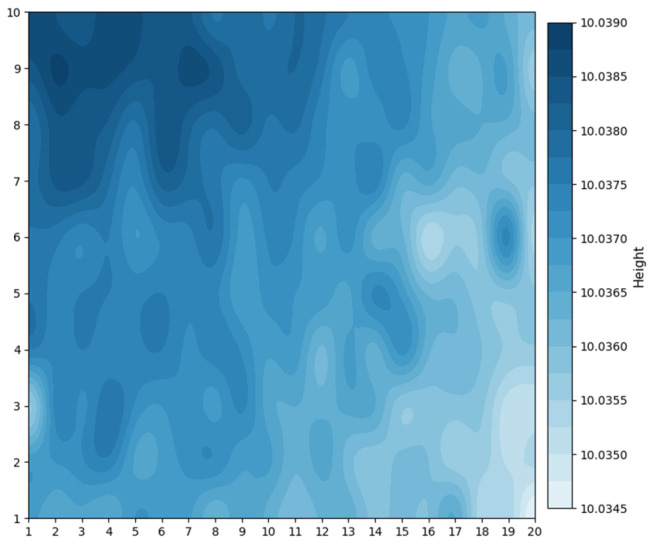
The flatness measurement results after polishing.

During the polishing process, the contact between the central region of the sample and the polishing slurry was relatively insufficient. To ensure thorough polishing in the central region, the edge of the sample was often over-polished, causing the polishing slurry to corrode the edges of the copper structure and, ultimately, resulting in a linewidth difference between the center and edge regions. By controlling the grinding process to make the central region of the sample 5 μm higher than the edge region, we achieved simultaneous and effective polishing across the entire sample during the polishing process, as shown in Figure 13. The polishing slurry was silica sol, and a polyurethane polishing pad was adopted. The rotation speed was set at 60 rpm with a slurry feed rate of two drops per second. Since polishing also thinned the sample, the 5 μm reserved height could balance the polishing uniformity and the final height consistency. The target value of height after grinding and polishing was 85 μm. Multi-point matrix thickness measurements were performed using the laser mode of a CNC Video Measuring System (Model VRM-H3030). A total of 20 points were collected in the transverse direction with a spacing of 3 mm, and 10 points in the longitudinal direction with a spacing of 6 mm, yielding a total of 200 measurement points. These data were used to reflect the surface flatness after grinding and polishing. The measurement results are shown in Figure 14, and the flatness was within 4 μm. As this value is obtained over a large area of the copper substrate, the flatness will be further improved for the region corresponding to each individual structure.

### 3.5. Conformal Resist Removal

The RD-2150 resist was removed using a two-step process combining solvent soaking and plasma etching. The plasma treatment was performed on a MA3000D plasma system (MUEGGE GmbH, Reichelsheim, Germany). First, the sample with photoresist was immersed in the Remover PG, followed by water bath heating at 60 °C for 3–5 h to swell and remove part of the photoresist. The residual resist was then removed by plasma etching. The etching parameters were set as follows: O_2_, CF_4_, and N_2_ flow rates of 1200 sccm, 70 sccm, and 70 sccm, respectively; process temperature of 60 °C; chamber pressure of 450 mTorr; input power of 1200 W; and processing time of 1.5 h.

**Figure 15:**
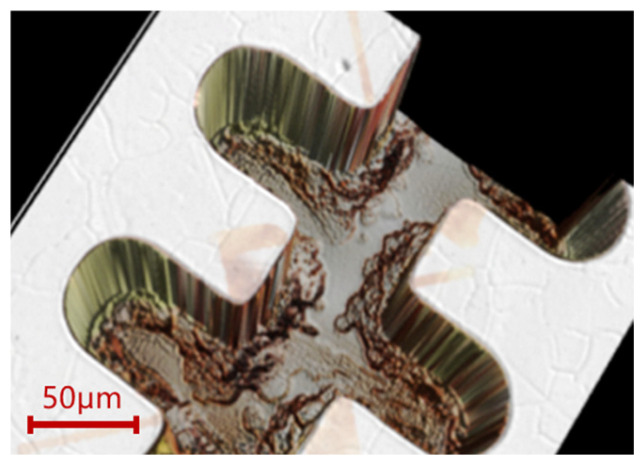
Morphological distortion at the bottom of the structure after resist removal.

**Figure 16:**
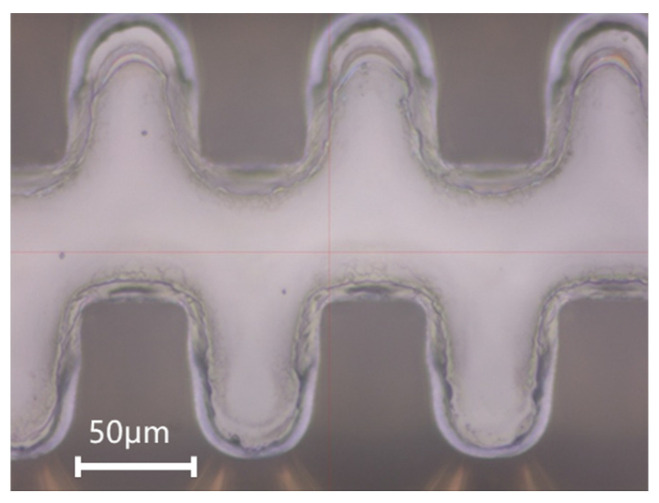
Damaged bottom morphology of the photoresist after development.

In the experiment, morphological distortion was always observed at the bottom of the structure after resist removal. When we used a 3D laser measuring microscope to image the morphology, a non-uniform step with a depth of about 4 μm was found at the bottom edge region, as shown in Figure 15. By tracing back the experimental process, this non-uniform step was an unwanted copper structure formed during the electroforming process. The edge region of the bottom resist was damaged and incomplete before electroforming, leading to the absence of the photoresist mold in that region during electroforming. We peeled the photoresist from the substrate after development, and the bottom morphology of the photoresist is shown in Figure 16. To prevent resist cracking during the post-exposure bake process, we set the holding time at the maximum baking temperature to only 25 min. Although 55 °C for 10 min and 75 °C for 25 min allowed the photoresist to adhere stably to the substrate with accurate top linewidth, there was still an issue of insufficient cross-linking at the bottom edge region, leading to partial dissolution of the resist during development. We further optimized the post-exposure bake parameters by reducing the heating rate from the original 10 °C/min to 1 °C/min during the process in temperature ramping from 55 °C to 75 °C. This process was employed to further crosslink the photoresist, ensuring the integrity of the photoresist after development.

**Figure 17:**
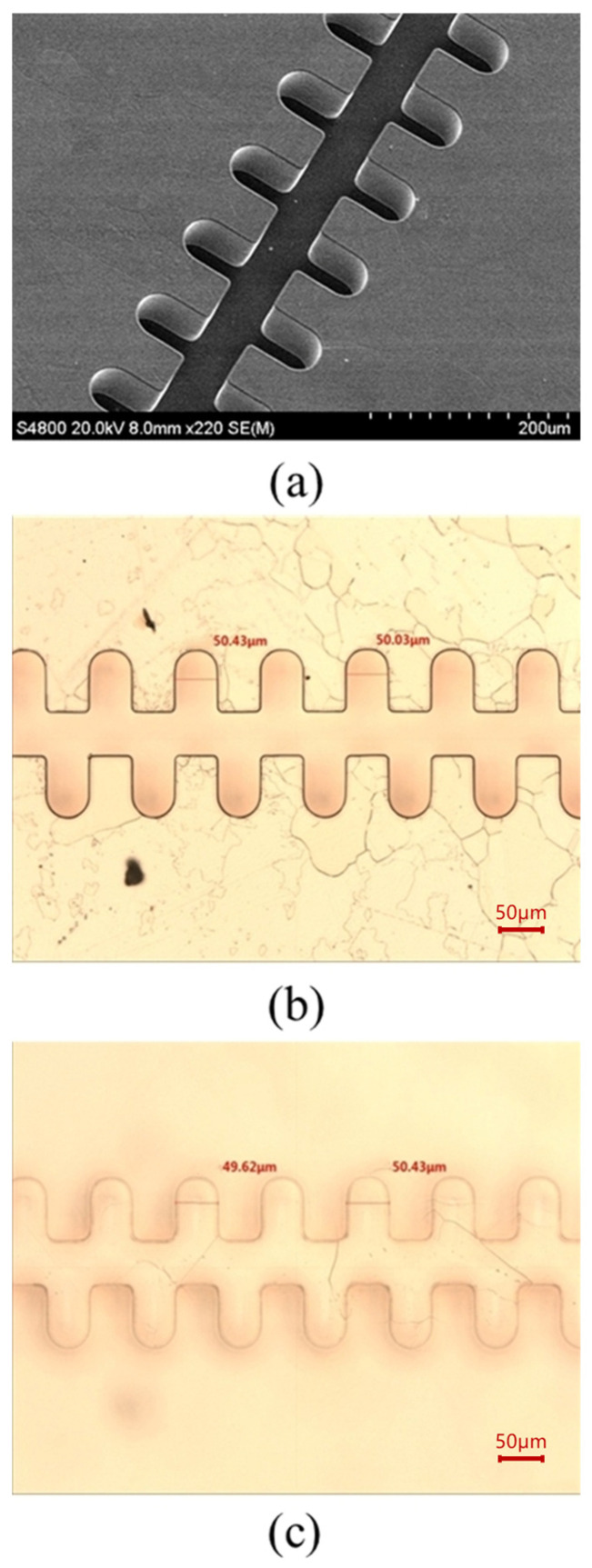
Fabricated 1.1 THz staggered double-grating slow wave structures. (**a**) SEM micrograph of the structure overall profile; (**b**) Optical micrograph of the top view; and (**c**) Optical micrograph of the bottom view.

After 1.5 h of plasma etching, the photoresist was completely removed, and conformal stripping with excellent sidewall verticality of the structure was realized. The sample was then subjected to hydrogen annealing at 850 °C for 30 min. No blistering or structural deformation was observed after annealing, indicating that the electroformed copper structure has excellent high-temperature stability. The corresponding SEM image (S-4800, Hitachi, Tokyo, Japan) and optical micrograph (MM-400, Nikon, Tokyo, Japan) are presented in Figure 17.

## 4. Results and Analysis

### 4.1. Dimensional Accuracy Measurement and Analysis

**Figure 18:**
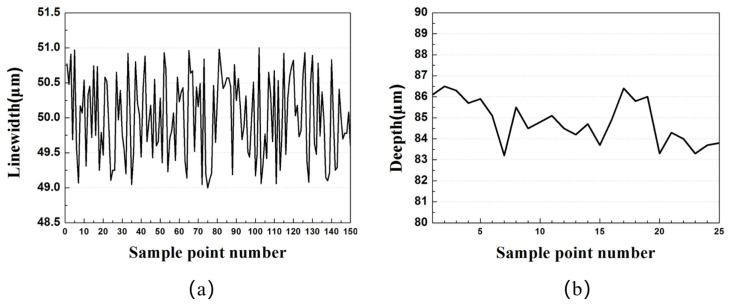
Linewidth and height measurement of a half-waveguide. (**a**) Linewidth measurement; (**b**) Height measurement.

The fabricated 1.1 THz staggered double-grating SWS consists of 150 periods. The linewidth of each period was automatically measured using a CNC video measuring system (VRM-H3030, Nikon, Tokyo, Japan; in optical mode) with displacement set according to the structural period. The measured linewidth varies from 49 μm to 51 μm, showing good agreement with the designed value of 50 μm, which corresponds to a linewidth accuracy of ±1 μm, as shown in Figure 18a. The dataset yields a mean value of 50.0074 and a standard deviation of 0.591217, with no discernible systematic bias. The linewidth demonstrates exceptional uniformity, which is attributed to the close contact between the mask and photoresist during photolithography. The integer components are precise, and minor decimal variations are primarily ascribed to instrumental measurement errors.

For the height measurement, 25 sampling points were uniformly arranged along the SWS. The total length of the fabricated SWS is 2 mm. The actual groove depth was calculated by the difference between the absolute heights of the structure’s top and bottom near each sampling point, which was measured via the laser mode of the same VRM-H3030 system. The designed half-waveguide height (h/2) is 85 μm, with the measured values ranging between 83 μm and 87 μm, corresponding to a depth accuracy of ±2 μm, as shown in Figure 18b. The dataset has a mean value of 84.852 and a standard deviation of 1.048459. A marginal decreasing trend in the overall height is observed with the shift of measurement points, which reflects the flatness of the substrate or polished surface in the corresponding region. Statistical analysis results for both the linewidth and height are tabulated in Table 2.

**Figure 19:**
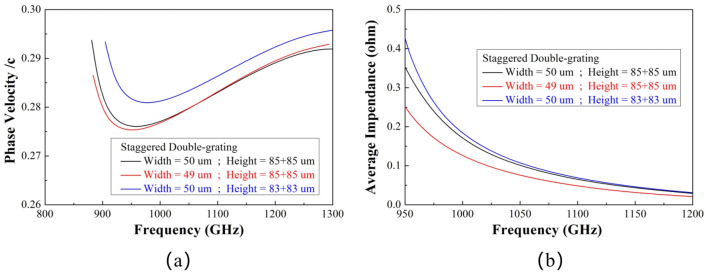
Phase velocity variation and coupling impedance variation induced by fabrication dimensional deviation. (**a**) Phase velocity variation; (**b**) Coupling impedance variation.

The influence of 1 μm linewidth deviation and 2 μm height deviation on the performance of the SWS is illustrated in Figure 19. As shown by the red curve, the linewidth deviation does not induce a significant change in the structural dispersion. The influence is mainly concentrated in the low-frequency range near 900 GHz, and the synchronization between the electron beam and the electromagnetic wave around the operating frequency of 1.1 THz is still well satisfied. However, the linewidth deviation causes a 21% reduction in the coupling impedance of the SWS, which will degrade the final output power of the device. For the case of height deviation (blue curve), the synchronous operating voltage of the structure increases, while the dispersion characteristic remains unchanged. Note that the worst-case scenario (i.e., a total height deviation of 4 μm) is considered here, which leads to an approximate voltage rise of 600 V. In contrast, the coupling impedance increases slightly under this condition, which will compensate for the output power degradation of the device.

### 4.2. Sidewall Verticality

**Figure 20:**
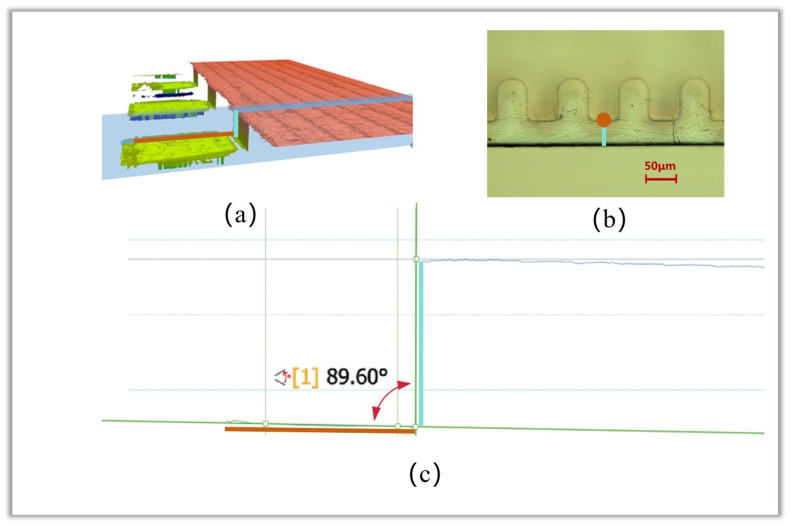
Sidewall verticality measurement results. (**a**) Three-dimensional morphology at the sampling position; (**b**) Top view of the structure; (**c**) Sidewall verticality fitting result.

To characterize the sidewall verticality of the fabricated staggered double-grating SWS, we cut the SWS to expose its cross-section. The cutting position was set to be nearly parallel to the electron beam tunnel of the SWS and located inside the tunnel. Sidewall verticality was characterized using a Sensofar optical profilometer. The sample was mounted at a tilt angle to fully expose the target sidewall. First, high-precision three-dimensional (3D) topographic data of the sidewall-adjacent region were acquired via the confocal scanning mode. Subsequently, two-dimensional (2D) height profiles were extracted along a predefined direction from the 3D topographic dataset. Finally, geometric fitting was performed on the profile points to calculate the sidewall angle. The positions marked in orange and blue in Figure 20a–c fully correspond. The included angle between the orange and blue lines is defined as the sidewall verticality of the fabricated structure. To facilitate direct positional alignment with Figure 20a,b, the fitted lines in Figure 20c are bolded in orange and blue, respectively. Figure 20b shows the top view of the structure without tilt mounting, where the red line corresponds to a single point at this viewing angle. Sidewall verticality measurements were performed at four independent positions on the same structure. The raw measurement results, mean value, and standard deviation are summarized in Table 3. The mean measured sidewall verticality is approximately 90.1°, with a standard deviation of approximately 0.5°, verifying excellent consistency of the developed microfabrication process.

### 4.3. Surface Roughness

**Figure 21:**
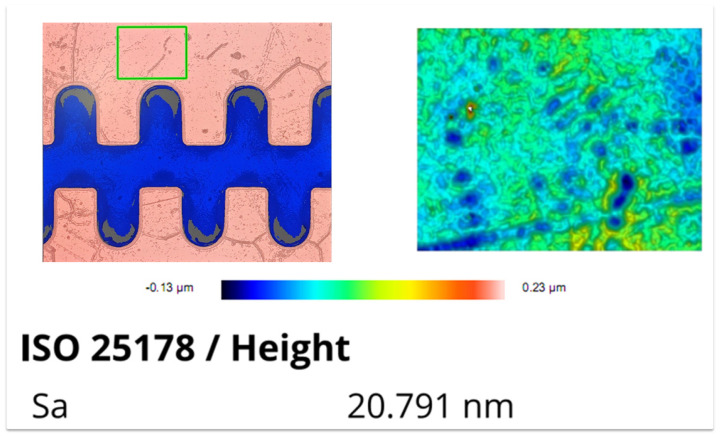
Measurement result of top surface roughness.

**Figure 22:**
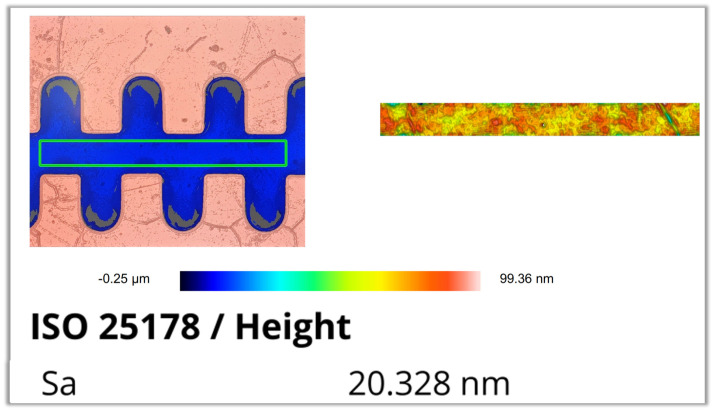
Measurement result of bottom surface roughness.

**Figure 23:**
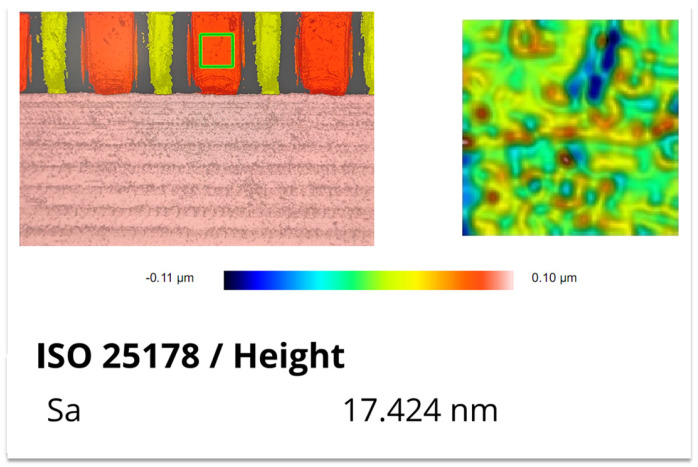
Measurement result of sidewall roughness.

The surface roughness of the fabricated 1.1 THz staggered double-grating SWS was characterized using a Sensofar optical profilometer (SENSOFAR TECH S.L., Terrassa, Spain). In consideration of the actual measurement precision of the instrument, the measured results were rounded to the nearest integer. The measured roughness is 21 nm for the top surface (sampling area: 80 μm × 60 μm), 20 nm for the bottom surface (sampling area: 300 μm × 30 μm), and 17 nm for the sidewall (sampling area: 30 μm × 30 μm). The corresponding test results and sampling positions are presented in Figure 21, Figure 22, and Figure 23, respectively.

## 5. Conclusions

We have demonstrated a UV-LIGA technology to microfabricate 1.1 THz staggered double-grating SWSs for TWTs in this article. The dimensional accuracies of the height and width of the structures are ±2 µm and ±1 µm, respectively. The surface roughness was within 21 nm. The sidewall verticality of the structures is 90.1°. This work has significantly optimized critical parameters, including dimensional accuracy, uniformity, and sidewall verticality. The successful fabrication of 1.1 THz slow-wave structures using UV-LIGA technology demonstrates that high-frequency structures produced by a UV-LIGA process are applicable to the development of higher-frequency vacuum electronic devices, laying a solid foundation for the advancement of terahertz devices. Currently, we are fabricating input and output circuits with tapered depth or stepped structures by combining mechanical machining and UV-LIGA technology, to facilitate the subsequent measurement of the S-parameters for electromagnetic characterization.

## Figures and Tables

**Table 1:** Dimensions of the 1.1 THz staggered double-grating SWS.

Parameter	Designed (μm)	Fabrication (μm)
Height (*h*)	85 × 2	85 ± 2
Width (*w*)	50	50 ± 1
Period (*p*)	100	100 ± 1
Width of the tunnel (*a*)	50	50 ± 1

**Table 2:** Statistical analysis of linewidth and height.

Parameter	Linewidth (μm)	Height (μm)
Mean	50.0074	84.852
Standard deviation	0.591217	1.048459
Error bar	±0.591217	±1.048459

**Table 3:** Raw data, mean value and standard deviation of the sidewall roughness measurements.

Position 1	Position 2	Position 3	Position 4	Mean	Standard Deviation
89.60°	90.70°	90.33°	89.80°	90.1°	0.5°

## Data Availability

The original contributions presented in this study are included in the article. Further inquiries can be directed to the corresponding author.
